# AIDS cases in Ottawa: A review of simultaneous HIV and AIDS diagnoses

**DOI:** 10.1111/phn.13065

**Published:** 2022-03-19

**Authors:** Lauren Orser, Patrick O'Byrne, Dave Holmes

**Affiliations:** ^1^ University of Ottawa School of Nursing Ottawa Canada; ^2^ Ottawa Public Health Sexual Health Clinic Ottawa Canada

**Keywords:** AIDS, AIDS‐related opportunistic infections, HIV, risk factors

## Abstract

**Objectives:**

In Canada, HIV diagnoses continue unabated, with many of these cases being identified at a late stage of infection. While current public health surveillance data does not capture timing of diagnoses, locally, we identified a number of patients concurrently diagnosed with AIDS and HIV.

**Design:**

To understand the key characteristics, presenting symptoms, and risk factors associated with an AIDS diagnosis, we undertook a prospective chart review of HIV and AIDS diagnoses in Ottawa, Canada.

**Sample:**

Sixty seven charts of persons diagnosed with HIV and AIDS between 2015 and 2021 were reviewed.

**Measurements:**

Data were analyzed using descriptive statistics.

**Results:**

Results show some inconsistencies regarding HIV risk factors identified in published literature compared to those for persons diagnosed with AIDS in this study. Namely, patients in this review were more likely to be male, Black (from HIV‐endemic regions), and heterosexual, and were diagnosed at critical stage in infection (total average CD4+ count of 92.9 cells/mm^3^) with 44.8% of patients concurrently diagnosed with one or more AIDS‐related opportunistic infections.

**Conclusions:**

The findings can be applied to strengthen HIV screening efforts in primary care settings, particularly among patients who present with persistent symptoms or illnesses related to chronic HIV infection. Additional considerations should be made for public health nurses to provide counseling and linkage to HIV testing/prevention services for patients at the time of an STI or Tuberculosis diagnosis and to increase AIDS‐specific data collection.

## 1 BACKGROUND

Despite the availability of free HIV testing services through various clinical and peer‐based settings across Canada, many persons continue to be diagnosed with HIV in a late disease stage, which is defined as occurring over 5 years from HIV acquisition (O'Byrne & Orser, 2019; Markwick et al., 2014; Muelas Fernandez et al., 2020; Public Health Agency of Canada [PHAC], 2019). While advancements in HIV treatment can restore most immune cell function, some opportunistic infections can cause permanent sequelae or death (O'Byrne & Orser, 2020; Poorolajal et al., 2016). This situation is more pronounced in persons who have progressed to AIDS, which, in Canada, is defined as having a CD4+ cell count of < 200 cells per cubic millimeter (cells/mm^3^) and/or the presence of AIDS‐related opportunistic infections (PHAC, 2016). Table 1. In addition, late diagnoses present a public health concern for potential onward transmission, given the usually high HIV viral load that accompanies an untreated infection. While most current evidence does not identify any transmissions involving persons with undetectable HIV viral loads (defined by most evidence as < 200 cells/ml), the HIV viral load in persons with chronic infections before treatment averages 205,862 copies/ml (range: 75,700–537,471) (Eisinger et al., 2019; Selik & Linley, 2018).

**TABLE 1:** AIDS‐defining conditions (Public Health Agency of Canada, 2019)

Opportunistic Infections
• Bacterial pneumonia, recurrent • Candidiasis (esophageal, bronchi, trachea or lungs) • Cervical cancer, invasive • Coccidioidomycosis (disseminated or extrapulmonary) • Cryptococcosis (extrapulmonary) • Cryptosporidiosis (chronic intestinal) • Cytomegalovirus disease (other than liver, spleen, nodes) • Cytomegalovirus retinitis (with loss of vision) • Encephalopathy, HIV‐related (dementia) • *Herpes simplex* virus (chronic ulcers or bronchitis, pneumonitis or esophagitis) • Isosporiasis, chronic intestinal • Kaposi sarcoma • Lymphoma (Burkitt, immunoblastic, primary in brain) • *Mycobacterium avium complex* or *M. kansasii* (disseminated or extrapulmonary) • *Mycobacterium* of other species (disseminated or extrapulmonary) • *Mycobacterium tuberculosis* (pulmonary, disseminated or extrapulmonary) • *Pneumocystis jiroveci* pneumonia • Progressive multifocal leukoencephalopathy • *Salmonella* septicemia, recurrent • Toxoplasmosis of brain • Wasting syndrome due to HIV

The importance of understanding such late diagnoses for individual and population health outcomes, however, is lost in current HIV surveillance in Canada. Current public health estimates do not differentiate between those who are diagnosed with recent (acute) or chronic (late) HIV infections (Haddad et al., 2019). In Ottawa, a retrospective review of HIV surveillance data from 2011 to 2014 revealed that 44% of HIV local cases were diagnosed late in their infection, the majority of whom were older men, women from HIV‐endemic regions, and persons with symptoms of chronic HIV infection (Friedman et al., 2017). Building on these findings, we completed a secondary review of the surveillance data for persons classified as having AIDS from 2015 to 2021 in Ottawa, where 67 cases were identified; 62 of which were new diagnoses. These findings are of interest to help guide decision‐making around HIV testing, to identify late HIV infections prior to progression to AIDS, and to help public health units strengthen HIV prevention efforts.

## 2 METHODS

This review occurred in Ottawa, Ontario, a city of approximately 1 million residents. Local public health surveillance data estimates an average of 55 HIV diagnoses per annum in Ottawa, the majority of which are among gay, bisexual, and other men who have sex with men (gbMSM), persons from HIV endemic regions, and persons who use injection drugs (Friedman et al., 2017).

In Ontario, Ministry of Health guidelines (2019a) indicate that all public health units must complete follow‐up with, and collect information about, newly reported cases of HIV and other sexually transmitted infections (STIs). This information is captured in patients’ files and transcribed into the *Integrated Public Health Information System* (iPHIS) as part of local monitoring and surveillance (Public Health Ontario [PHO], 2021a). Following what we observed as an increase in the number of patients being diagnosed in a late stage of HIV infection, we began tracking AIDS diagnoses in Ottawa on January 1, 2015 and continued until December 31, 2021. A prospective chart review was completed involving patients diagnosed with AIDS from 2015 to 2021 in Ottawa using mandatory Ministry of Health (2019b) surveillance items collected from iPHIS (i.e., demographic information, date of diagnosis, presence of symptoms, and infection specific risk factors). Additional data were obtained from a manual review of patients’ corresponding public health charts.

### 2.1 Data collection

For inclusion, patients were required to have (1) a positive HIV serology report (third or fourth generation) indicating positive HIV 1/2 Antibody/Antigen screen and confirmatory HIV antibody on Western Blot, Geenius, or P24 Antigen, (2) HIV diagnosis or follow‐up within Ottawa, and (3) AIDS defining criteria, including a CD4+ T‐cell count of ≤200 cells/mm^3^ and/or diagnosis of an AIDS‐defining illness (per Table 1) (PHAC, 2016; Ontario Ministry of Health, 2019b). Data extraction was completed in February 2021 and updated in February 2022. Cases were reviewed by two researchers (L. O. and P. O. B.) to ensure they met the foregoing inclusion criteria. Where discrepancies were identified in classifications, cases were discussed by the research team to determine inclusion into the study. Per mandatory public health surveillance, information was compiled on patient demographics, immigration status, HIV diagnosis, symptoms, CD4+ count/AIDS‐related illness, STI diagnoses (past and present), and risk factors (Ontario Ministry of Health, 2019a; 2019b). All data were analyzed using descriptive statistics, looking at the total sample and differences between males and females.

### 2.2 Ethics

Approval for this study was obtained by the Ottawa Public Health ethics committee following a low‐risk score on the Public Health Ontario (2019) risk screening tool. Based on this score and the fact this review involved mandatory public health surveillance data, formal ethics board approval and individual consent from patients was not required. Risk of harm to patients was also considered negligible based on findings from the risk screening tool.

## 3 RESULTS

### 3.1 Patient characteristics

From January 1, 2015 to December 31, 2021, 67 cases of AIDS were diagnosed in Ottawa. AIDS case counts compared to the total number of reported HIV diagnoses in Ottawa during this period are as follows: 4/47 (8.5%) cases in 2015, 11/65 (16.9%) cases in 2016, 13/62 (20.9%) cases in 2017, 12/75 (16.0%) cases in 2018, 7/35 (20.0%) cases in 2019, 11/37 (29.8%) cases in 2020, and 9/46 (19.5%) cases in 2021 (PHO, 2021b). Sixty‐two of the 67 total cases were new diagnoses, meaning these patients were previously unaware of their HIV‐positive status and were diagnosed with HIV and AIDS concurrently. Of these AIDS cases, 76.11% (*n* = 51) were male and 23.9% (*n* = 16) were female. None identified as trans. The average age of all patients diagnosed with AIDS was 49.5 years old (range: 18–80 years). By gender, the average age among males was 52.0 years and among the average age was 44.7 years. For ethnicity, 53.7% of patients identified as Black (*n* = 36) and 38.8% were White (*n* = 26). When patient characteristics were examined according to sex and ethnicity, 81.3% (*n* = 13/16) of AIDS diagnoses among females were in Black women and 45.1% (*n* = 23/51) of diagnoses among males were in Black men; an additional 47.1% were in White men (*n* = 24/51). When concurrent HIV/AIDS diagnoses were grouped according to population‐specific risk factors, 50.7% (*n* = 34) were African, Caribbean, or Black (ACB), 26.8% (*n* = 18) were heterosexual from non‐HIV endemic regions, 11.9% (*n* = 8) were gbMSM, and 5.9% (*n* = 4) were persons who use drugs. We were unable to classify population‐specific risk factors for three patients (Table 2).

**TABLE 2:** Demographic characteristics of AIDS patients by sex

	**Males**		**Females**	
	**n**	**%**	**n**	**%**
Sex	51	73.6	16	26.3
Age	52.0 years	‐	44.7 years	‐
Ethnicity				
Black	23	45.1%	13	81.3%
White	24	47.0%	<5	‐
Other	<5	‐	<5	‐
Population				
ACB	21	41.2%	15	81.3%
Heterosexual (non‐endemic)	16	31.4%	<5	‐
gbMSM	7	13.7%	n/a	‐
People who use drugs	<5	‐	<5	‐
Unknown	<5	‐	0	‐
Country of birth				
Born in Canada	24	47.1%	<5	‐
Born outside of Canada	27	57.1	15	93.8%
Time since immigration	18.5 years	‐	9.7 years	‐
Range	(2 to 56 years)		(2 to 16 years)	

### 3.2 Immigration status

Over one third (*n* = 26) of participants were born in Canada. The remaining two thirds (*n* = 41) were born outside of Canada, of whom 85.3% (*n* = 35/41) were born in HIV‐endemic regions–primarily Africa and the Caribbean. Among the 13 Black females, all were born outside of Canada; for the 23 Black men, all but one was born in an HIV‐endemic region. An additional 8.9% (*n* = 6) patients were born in Europe or South East Asia. Among those born outside of Canada, 17 completed HIV screening upon arrival (classified as within 1 year), whereas 20 patients had been living in Canada for longer–averaging 17.2 years for all patients (range: 2–56). Among males, the average time since immigration was 18.5 years (range: 2–56 years) and for females the average time since immigration was 9.7 years (range: 2–16 years). Information on time since immigration was not available for four patients. Table 2.

### 3.3 STBBI history

Among patients in this review, 20.9% (*n* = 14) had a previous STI diagnosis: three had hepatitis C, three had hepatitis B, two had syphilis, one had chlamydia, three had gonorrhea, and three patients had multiple episodes of STIs. Among patients with a previous STI diagnosis, 92.9% (*n* = 13/14) were male, of whom the majority were White and reported sex with same sex partners, sex with opposite sex partners, or injection drug use. Notably, 28.4% (*n* = 19) of patients were diagnosed with a secondary infection (STI or tuberculosis) at the same time as their HIV diagnosis. Of the concurrent diagnoses made during this review period, six were syphilis, two were hepatitis C, and two were hepatitis B. The majority of concurrent infections were identified among males (*n* = 14), of whom, six were White and six were Black. One patient was diagnosed with multiple STIs concurrent to their HIV/AIDS diagnosis. Eight patients (six males and two females) were diagnosed with latent tuberculosis (Tb). The majority of Tb infections were in Black patients from HIV‐endemic regions (*n* = 5/8).

### 3.4 HIV testing

We identified five primary locations of HIV/AIDS diagnosis among our patients: 32.8% (*n* = 22) were during in‐patient hospital admissions, 23.8% (*n* = 16) were in specialist clinics, 19.4% (*n* = 13) were in primary care and walk‐in settings,16.4% (*n* = 11) were during immigration medical examination, and 7.5% (*n* = 5) were in emergency or urgent care departments. Nearly 30% (*n* = 19) of patients had a documented or reported history of a negative HIV result, ranging from one to 20+ years prior (average 8.3 years). Most patients (*n* = 33), however, did not recall their last negative HIV test and an additional nine patients reported no prior HIV screening. Persons without prior HIV testing (i.e., unknown history or never tested) accounted for 71.6% of concurrent HIV and AIDS diagnoses during the study period, of whom 75% (*n* = 36/48) were males and were fairly evenly distributed between White and Black men (17 and 19 cases, respectively) and 25% (*n* = 12/48) were female, most notably, Black women from HIV endemic regions (*n* = 11/12). Five patients were previously diagnosed with HIV, none of whom was on HIV treatment at the time of this review.

### 3.5 Presenting symptoms

Of the 67 patients diagnosed with AIDS, nine presented without symptoms (five males and four females). The other 58 patients presented with multiple symptoms affecting multiple systems. A complete list of symptoms can be found in Table 3. Some of the predominant symptoms associated with an AIDS diagnosis in this review included: weight loss greater than 10% of body‐weight and loss of appetite (*n* = 34/58 or 58.6%); unexplained fever and malaise (*n* = 25/58 or 43.1%); dyspnea or dry cough (*n* = 25/58 or 39.7%); recurrent oral thrush (*n* = 21/58 or 36.2%); and chronic fatigue and lethargy (*n* = 20/58 or 34.5%).

**TABLE 3:** HIV/AIDS symptoms among all patients diagnosed with AIDS in Ottawa

**Symptom and Category**	**N = 227**	***%* / *N* % / n**
***General***	***n = 90***	***39.6%***
Weight loss >10% of body weight or loss of appetite	30	33.3%
Chronic fatigue, lethargy or malaise	24	26.7%
Fever	20	22.2%
Lymphadenopathy	9	10%
Chronic night sweats	7	7.8%

***Dermatologic***	***n = 40***	***17.6%***
Thrush	18	45%
Oral, genital, or skin lesions	14	35.0%
Herpes zoster or increased severity of herpes simplex virus (HSV)	8	20.0%

***Neurologic***	***n = 35***	***15.4%***
Cognitive decline or decreased level of consciousness	10	28.6%
Headache	6	17.1%
Generalized weakness	5	14.2%
Hearing or vision loss	4	11.4%
Dizziness	4	11.4%
Ataxia, paresthesia, myelopathy, or osmotic demyelination	4 (1 each)	11.4%
Meningitis or hydrocephalus	2	5.7%

***Respiratory***	***n = 34***	***14.9%***
Dyspnea or dry cough	25	73.5%
Pneumonias	9	26.5%

***Abdominal***	***n = 21***	***9.3%***
Chronic diarrhea	11	52.4%
Acute kidney injury, renal failure, or elevated lactate dehydrogenase (LDH)	7	33.3%
Abdominal pain	3	14.3%

***Cardiac***	***n = 7***	***3.1%***
Pericardial effusion, endocarditis, myocarditis, or deep vein thrombosis	4 (1 each)	57.1%
Tachycardia	2	28.6%
Cardiac arrest	1	14.2%

### 3.6 AIDS diagnosis

For 37 of the 67 patients in this review, the diagnosis of AIDS was made based on the report of CD4+ count < 200 cells/mm^3^. The total average CD4+ count for all patients was 92.9 cells/mm^3^ (range 3–335 cells/mm^3^). AIDS diagnosis by CD4+ count accounted for 57.1% (*n* = 24/42) of the total cases among males and 60% (*n* = 9/15) of the total cases among females. For the remaining 30 patients who were not diagnosed by CD4+ count alone, AIDS diagnosis was made based on the presence of one or more AIDS‐related opportunistic infections, regardless of CD4+ count. In this review, 73.3% (*n* = 22) of patients had a single diagnosis, while 26.6% (*n* = 8) of patients were found to have two or more opportunistic infections at the point of AIDS diagnosis. These illnesses were as follows: *Pneumocystis jirovecii* pneumonia (*n* = 16), Kaposi's sarcoma (*n* = 6), Toxoplasmosis of brain (*n* = 4), Cytomegalovirus (*n* = 4), Candidiasis of the esophagus, bronchi, trachea or lungs (*n* = 3), *Salmonella* septicemia (*n* = 1), *Herpes simplex*: chronic ulcer(s) (*n* = 1), Progressive multifocal leukoencephalopathy (*n* = 1), Cryptococcosis (*n* = 1), and *Mycobacterium* of unidentified species (*n* = 1). To the best of our knowledge, most patients diagnosed with AIDS in this review were initiated on antiretroviral treatment; however, four patients were admitted to hospital at the time of HIV diagnosis and died from AIDS‐related complications during admission.

## 4 DISCUSSION

The results from our prospective review of public health surveillance data of patients diagnosed with AIDS in Ottawa present some interesting findings in terms of patient characteristics and presenting illness. In particular, over three‐quarters of patients diagnosed with AIDS in this review were male. We also identified differences in the proportion of heterosexual HIV exposures compared to Canadian estimates. While in Canada in 2019, the proportion of new HIV diagnoses among persons with opposite sex partners was 28.3% (Haddad et al., 2019), in our review heterosexual HIV transmission accounted for 74.6% (*n* = 50) of those diagnosed with AIDS. Building on this, we identified that persons born in HIV‐endemic regions were more likely to be affected by AIDS, accounting for two thirds of all heterosexual HIV risk exposures in this review compared to the Canadian estimates of heterosexual transmission from HIV‐endemic regions of 9.2% (Haddad et al., 2019). Similar discrepancies were identified in terms of HIV exposures among individuals with same sex partners from non‐HIV endemic regions, who accounted for 8.2% of the total HIV cases diagnosed in Canada in 2019, compared to 26.9% of patients diagnosed with AIDS in this review (Haddad et al., 2019).

These findings also highlight several key recommendations for primary care providers, including nurse practitioners and registered nurses, as well as public health units and nurses. First, for primary care providers, while belonging to a group known to be affected by HIV can be an indicator for testing, nonmembership should not preclude such testing, particularly for patients who present with chronic HIV symptoms (Haddad et al., 2019; PHO, 2021a; Centers for Disease Prevention & Control [CDC], 2006). The fact that nearly one‐quarter of all patients diagnosed with AIDS in this review were (primarily) White heterosexual males demonstrates that designation to a priority group does not rule out HIV as the etiology of chronic symptoms. When patients present with symptoms suspicious of chronic HIV infection, HIV should be considered as a differential diagnosis, regardless of ethnicity, country of birth, or reported risk practices. In addition, HIV screening should be considered among patients with a past history of STIs, including chlamydia, gonorrhea, syphilis, hepatitis B, and hepatitis C (Hull et al., 2017; Pathela et al., 2015). That 14 patients in this review diagnosed with AIDS had a previous STI diagnosis (nearly three‐quarters of whom belonged to a group at elevated risk for HIV) and did not complete HIV testing at the same time highlights missed opportunities for early HIV diagnosis.

Considering the high proportion of heterosexual AIDS transmissions, it is also important to increase HIV prevention and screening efforts among heterosexual women–particularly those who were born in HIV‐endemic regions and/or who use injection or inhalation drugs. In this review, ACB females born outside of Canada were found to be disproportionately affected by AIDS compared to ACB males. While AIDS diagnoses among women in this review were the minority (23.9% of total sample), this group did account for 11–36% of concurrent HIV/AIDS cases reported each year during the study period (with the exception of 2015). This finding aligns with findings from the Ontario HIV Epidemiology and Surveillance Initiative (2017), which estimates one in five new HIV diagnoses in Ontario are now among women, but differs slightly from recent Ottawa data which showed that new HIV diagnoses were higher among women compared to men in 2019 (PHO, 2021b). More work needs to be done to better understand HIV/AIDS‐related risk indicators among women, particularly those from racial and ethnic minority groups to improve current diagnostic and HIV prevention efforts for this group.

Second, the high number of ACB persons born outside of Canada who were diagnosed with AIDS in this review highlights the need for primary care providers (1) to ensure a documented HIV negative result on file, (2) complete HIV screening for those who are unaware of their HIV status, and (3) offer routine HIV testing for ACB persons who report recent travel to, or new sexual partners from, HIV‐endemic regions. This is an important consideration, as HIV screening only became mandatory on immigration medical exams in Canada in 2002; patients who immigrated before this time may not have been appropriately assessed or screened for HIV (CATIE, 2020). These recommendations are further supported by the 37.8% of patients in this review who reported having lived in Canada for a decade or more before being diagnosed with HIV, at which point their infection had progressed to AIDS. Moreover, it is possible that when HIV testing is done at the point of immigration, an HIV diagnosis could be missed if this test occurs within weeks of a possible risk exposure (i.e., when patients are still in the window period for HIV testing). Notably, half of the patients who died from AIDS‐related complications in this review were from ACB groups and had been living in Canada for more than 15 years, signaling that routine HIV screening should be done on all non‐Canadian born patients, in particular those who report recent travel to or immigration from HIV‐endemic areas or who report sexual partners with recent travel or immigration from these areas.

The third point of consideration for primary care providers is around assessment of chronic HIV symptoms. When patients present with unexplained and persistent symptoms, we suggest providers compare these symptoms to those suggestive of chronic HIV infection (PHAC, 2016). When possible overlap is identified, HIV screening should be considered, regardless of patients' assumed or reported risk factors for infection–and especially when patients report their HIV status as unknown. The foregoing point is supported by data from this review which found that 86.6% of patients diagnosed with AIDS had recurrent symptoms of chronic HIV‐infection and over 60% of AIDS diagnoses were made outside of primary care settings (e.g., in‐patient, specialist, and emergency departments) when illnesses had become particularly advanced.

In terms of recommendations for public health practice, we suggest public health nurses provide counseling on, and make recommendations for, HIV testing for persons from at‐risk groups who are diagnosed with an STI (e.g., chlamydia, gonorrhea, syphilis, hepatitis B, hepatitis C) and/or Tb. Of the 14 patients in this review who had a documented history of STIs, half reported having never completed HIV testing before or being unaware if this had occurred, and an additional five reported a prior negative HIV test 1–10 years prior. Considering the association between HIV acquisition and STIs, a diagnosis among persons who belong to groups with elevated HIV prevalence, such as ACB persons from HIV‐endemic regions, persons from Indigenous communities, gbMSM, people who use drugs, and trans men and women, should reflexively warrant counseling on HIV transmission and HIV testing and prevention options (Galvin & Cohen, 2004; O'Byrne et al., 2019; Tan et al., 2017). Moreover, additional efforts should be made to ensure HIV status is established for persons diagnosed with rectal gonorrhea and/or infectious syphilis and a referral for HIV pre‐exposure prophylaxis (PrEP) is offered to these individuals (when HIV‐negative status is established) given the known correlation between and these infections and an HIV diagnosis, particularly among gbMSM (CDC, 2021a; 2021b).

While it is possible that HIV infection had already occurred for a subset of patients in this review at the time of their documented STI diagnosis, recommendations for HIV testing by public health nurses could have led to earlier diagnosis and treatment initiation, and potential reduction in the negative individual and public health outcomes associated with advanced HIV infection (Goldschmidt & Chu, 2021). Conversely, if HIV infection had not yet occurred for these patients, it is possible that counseling on, and linkage to, routine screening and HIV prevention services (e.g., HIV pre‐ and post‐exposure prophylaxis) could have averted HIV infection in some cases. Similar recommendations for HIV/STI screening should also be considered for patients diagnosed with Tb. While data on Tb history prior to HIV/AIDS diagnosis was not captured in this review, the number of patients concurrently diagnosed with AIDS and Tb suggests this could be an indicator of HIV risk, particularly among patients who report a history of immigration from an HIV‐endemic region. This assertion aligns with data on HIV/AIDS and Tb coinfections, which indicates that “HIV is the most powerful risk factor predisposing *Mycobacterium tuberculosis* infection and progression to active disease” (Naing et al., 2013; Pawlowski et al., 2012, p.1). As such, it would be prudent for public health nurses involved in communicable disease management to also consider HIV/AIDS as an underlying factor in Tb diagnosis and follow‐up.

Finally, the ongoing rates of concurrent HIV and AIDS diagnoses in Ottawa revealed in this review highlight the need for increased data collection on AIDS cases by public health units. While information on HIV risk infection and risk factors is widely available in Ontario, data specific to AIDS is not readily accessible as, in Ontario, HIV and AIDS are classified and reported as unique infections (PHO, 2021b). As was uncovered in this analysis, patients concurrently diagnosed with HIV and AIDS had risk factors that varied from those typically associated with HIV (e.g., older White men). Increasing efforts to collect AIDS‐specific data according to national or provincial/state guidelines within public health departments could be a strategy to strengthen understanding of risk indicators of chronic HIV infection so as to better target HIV testing and prevention efforts for populations or subgroups found to be at elevated risk of AIDS.

### 4.1 Limitations

This review involved information from public health surveillance files in one Canadian city where access to HIV testing is free. Results could have differed had this review occurred in a city with a larger population or involving different population groups. For example, few patients in this review identified as persons who use drugs, and no one reported being from trans or Indigenous groups. In addition, HIV surveillance data are limited to information about diagnosis and does not capture information on treatment regimens, secondary health conditions, or retention in care. Moreover, HIV viral load tests are not reportable in Ontario, thus we could not report on viremic levels of patients with AIDS; however, viremic levels are not a defining component of an AIDS diagnosis in Ontario.

## 5 CONCLUSION

The findings from this review have useful implications for primary care providers in terms of the importance of completing routine HIV screening in patients who present with symptoms of chronic HIV infection–regardless of their designation to an HIV priority population group or self‐reported HIV risk behaviors. Furthermore, the high number persons diagnosed from ACB groups highlights the need to heighten the response among primary care providers for HIV prevention initiatives, including routine HIV screening, rapid antiretroviral treatment initiation, and HIV pre‐ and post‐exposure prophylaxis for those who are HIV‐negative or of unknown HIV status. Ideally, for all patient populations, considering HIV as an appropriate differential diagnosis can help practitioners identify the etiology of persons with chronic symptoms and infections and help decrease onward HIV transmission. Moreover, increasing reflexive offers for HIV testing and HIV prevention services by public health nurses to persons who belong to groups with elevated HIV prevalence who are diagnosed with STIs (particularly, rectal gonorrhea and infectious syphilis) and tuberculosis could help to identify HIV diagnoses prior to the development of AIDS, and in some cases, prevent HIV acquisition all‐together. Finally, increased efforts should made for public health units and departments to collect demographic and risk‐related data on patients diagnosed with AIDS, as these were found to differ from HIV‐specific data so as to HIV screening efforts.

## ETHICAL STATEMENT

This study involved a prospective chart review of the Ontario public health database patients diagnosed with AIDS at the time of HIV diagnosis. Ethics approval was obtained by the Ottawa Public Health ethics committee following a low‐risk score on the Public Health Ontario risk screening tool. Formal ethics board approval and individual consent from patients was not required for this study.

## CONFLICT OF INTEREST

We confirm the information contained in this manuscript has not been published elsewhere and is not currently under review with an alternate journal.

## Data Availability

The data that support the findings of this study are available from the corresponding author upon reasonable request.
